# Treatment of Brain Metastases: The Synergy of Radiotherapy and Immune Checkpoint Inhibitors

**DOI:** 10.3390/biomedicines10092211

**Published:** 2022-09-07

**Authors:** Jennifer K. Matsui, Haley K. Perlow, Rohit K. Raj, Ansel P. Nalin, Eric J. Lehrer, Rupesh Kotecha, Daniel M. Trifiletti, Shearwood McClelland, Kari Kendra, Nicole Williams, Dwight H. Owen, Carolyn J. Presley, Evan M. Thomas, Sasha J. Beyer, Dukagjin M. Blakaj, Manmeet S. Ahluwalia, Raju R. Raval, Joshua D. Palmer

**Affiliations:** 1 College of Medicine, The Ohio State University, Columbus, OH 43210, USA; 2 Department of Radiation Oncology, The Ohio State University Wexner Medical Center, Columbus, OH 43210, USA; 3 Department of Radiation Oncology, Icahn School of Medicine at Mount Sinai, New York, NY 10029, USA; 4 Department of Radiation Oncology, Miami Cancer Institute, Baptist Health South Florida, Miami, FL 33176, USA; 5 Department of Radiation Oncology, Mayo Clinic, Jacksonville, FL 32224, USA; 6 Departments of Radiation Oncology and Neurological Surgery, University Hospitals Seidman Cancer Center, Case Western Reserve University School of Medicine, Cleveland, OH 44106, USA; 7 Division of Medical Oncology, The Ohio State University Wexner Medical Center, Columbus, OH 43210, USA; 8 Department of Medical Oncology, Miami Cancer Institute, Baptist Health South Florida, Miami, FL 33176, USA

**Keywords:** brain metastases, radiotherapy, radiation therapy, systemic therapy, immune checkpoint inhibitors, immunoradiotherapy

## Abstract

Brain metastases are a devastating sequela of common primary cancers (e.g., lung, breast, and skin) and have limited effective therapeutic options. Previously, systemic chemotherapy failed to demonstrate significant benefit in patients with brain metastases, but in recent decades, targeted therapies and more recently immune checkpoint inhibitors (ICIs) have yielded promising results in preclinical and clinical studies. Furthermore, there is significant interest in harnessing the immunomodulatory effects of radiotherapy (RT) to synergize with ICIs. Herein, we discuss studies evaluating the impact of RT dose and fractionation on the immune response, early studies supporting the synergistic interaction between RT and ICIs, and ongoing clinical trials assessing the benefit of combination therapy in patients with brain metastases.

## 1. Introduction

Brain metastases are the most common brain tumor and frequently originate from primary lung cancer, breast cancer, and melanoma [1]. Brain metastases account for a disproportionately high percentage of morbidity and mortality among patients with cancer [2], with dismal 2- and 5-year survival rates of 8.1 and 2.4% after diagnosis [3]. There are an estimated 200,000 new brain metastases diagnoses per year, and this number is projected to increase as systemic treatment modalities and imaging techniques improve [4,5,6]. Despite the increasing prevalence of brain metastases, there are limited treatment options. While radiotherapy (RT) is a mainstay to treat brain metastases [7], systemic therapies have historically demonstrated limited ability to penetrate the blood–brain barrier (BBB). More recently, immune checkpoint inhibitors (ICIs) have emerged as a promising treatment option for patients with brain metastases [8]. Researchers are now interested in merging RT with immunotherapy agents to produce a synergistic effect. This review highlights promising ICIs in the treatment of brain metastases, the effects the RT dose and fractionation have on the immune system, studies evaluating RT and ICI synergy, the effects of ICI/SRS timing, and relevant ongoing clinical trials.

## 2. Brain Metastases Treatment Management

### 2.1. Local Therapy

Historically, local therapies such as RT and surgery played a key role in the management of brain metastases treatment. The seminal case series by Chao et al. was the first report describing the palliative benefit of WBRT for brain metastases [9]. Patchell et al. reported that the addition of surgery to WBRT improved local control (LC) and median overall survival (OS) [10]. Then in 1998, Patchell et al. reported that WBRT after surgery reduced recurrence and neurologic death compared with observation [11]. Despite the advantages of WBRT, treatment is associated with short- and long-term neurologic complications (e.g., leukoencephalopathy, cognitive decline) [12]. The concerns related to WBRT-induced toxicity led neuro-oncologists to seek alternative treatment strategies for brain metastases. More recently, stereotactic radiosurgery (SRS) has emerged as a more precise radiation modality that spares healthy brain tissue [13].

Chang et al. reported that patients treated with SRS alone experienced better neurocognitive outcomes than patients who received SRS plus WBRT [14]. In 2014, Yamamoto et al. conducted a prospective randomized trial to determine if patients receiving SRS with 5 to 10 brain metastases had non-inferior survival outcomes to patients with 2 to 4 brain metastases [15]. Median OS for patients with 2 to 4 lesions was equivalent to median OS for patients with 5 to 10 lesions (10.8 months in both arms), suggesting SRS may be an appropriate alternative to WBRT in patients with up to 10 brain metastases. In 2017, the NCCTG N107C/CEC·3 phase III trial compared outcomes of post-operative SRS with post-operative WBRT in patients with brain metastases [16]. This study found no difference in OS but worsened cognitive decline in the WBRT treatment group at 6 months (85% vs. 52%). Although there was not a significant survival difference between the two groups, local and distant brain control was worse in the SRS group. These findings suggested that patients with one to three brain metastases may experience durable local control with SRS and that SRS is a viable alternative to WBRT. NCCTG N0574 compared outcomes between patients with one to three brain metastases randomized to SRS plus WBRT or SRS alone [17]. The authors found the addition of WBRT led to a decline in immediate recall (31% vs. 8%), delayed recall (51% vs. 20%), and verbal fluency (19% vs. 2%). Although WBRT did not improve OS, there was greater intracranial tumor control at 12 months in the SRS plus WBRT arm (84.9% vs. 50.5%). Subsequently, the JCOG0504 phase III non-inferiority trial studied whether SRS alone was as effective as WBRT or WBRT plus SRS [18]. Although intracranial progression-free survival was longer in the WBRT arm, the median OS in both arms were equivalent (15.6 months). Furthermore, the Mini-Mental Status Examination (MMSE) score decline between the two groups was not significant, but grade 2 to 4 adverse events were higher in the WBRT arm. These findings suggested that SRS can be considered standard therapy for patients with four or fewer brain metastases.

Recognizing the toxicities associated with WBRT, research groups developed techniques to limit irradiation to the hippocampal dentate gyri and hypothesized that preserving the neural stem cells may prevent WBRT-induced cognitive toxicity [19,20]. In 2020, Brown et al. published a phase III trial (NRG Oncology CC001) comparing cognitive decline and survival outcomes in patients receiving hippocampal avoidance (HA) WBRT plus memantine or WBRT plus memantine [21]. The authors found that HA-WBRT plus memantine resulted in less executive function and learning/memory deterioration with no significant difference in intracranial PFS and OS. Based on these findings, patients with brain metastases planned for WBRT may benefit from HA-WBRT if there are no metastases in the hippocampal avoidance region.

### 2.2. Systemic Therapy

Common chemotherapies (e.g., cisplatin and paclitaxel) have been evaluated in clinical trials but have failed to demonstrate a significant benefit in patients with brain metastases [22,23,24]. Researchers have found that the BBB, efflux pumps, and the blood–tumor barrier may prevent the cytotoxic agents from reaching effective concentrations [25]. More recently, neuro-oncologists were able to identify molecular drivers for a variety of primary cancers that have a propensity of spreading to the brain (e.g., lung cancer, breast cancer, and melanoma). Understanding these key signaling pathways has led to the development of novel targeted treatments such as tyrosine kinase inhibitors (TKIs) and immune-related therapy that have demonstrated intracranial efficacy, especially in patients with asymptomatic brain metastases [26]. The successful utilization of systemic therapy will be discussed in greater detail below.

## 3. Immunotherapy in the Treatment of Brain Metastases

Despite the success of ICIs across various tumor types, patients with brain metastases have been excluded from ICI trials due to a limited CNS penetration and poor prognosis [27]. The central nervous system (CNS) was thought to be an immune-privileged site, but brain metastases were found to be surrounded by an inflammatory microenvironment, suggesting otherwise [28,29]. Previously, monoclonal antibodies were thought to be too large to cross the BBB, but some studies reported ICI efficacy in treating brain metastases [30]. This observed activity may be related to (1) leaky tumor neo-vessels and (2) anti-tumor T cells that may be primed and activated at extracerebral sites. Notable studies exploring the efficacy of ICI agents will be emphasized in this section.

### 3.1. Lung Cancer Brain Metastases

Lung cancer is the most common cause of brain metastases and is categorized as small cell lung carcinoma (SCLC) and non-small cell lung carcinoma (NSCLC). NSCLC is typically more chemo-resistant and has a high propensity for brain metastases [31]. This section will focus primarily on NSCLC studies.

Patients with NSCLC that have failed first-line treatment have historically had limited treatment options. In the search for effective therapies, researchers were interested in targeting the programmed death 1 (PD-1) receptor that is expressed on activated T cells. The PD-1 receptor engages with ligands PD-L1 and PD-L2, which are expressed by cancer cells [32]. This interaction inhibits T-cell activation, thus allowing tumor cells to escape immune system recognition [33].

Nivolumab is an IgG4 PD-1 ICI antibody that inhibits PD-1 signaling, therefore restoring anti-tumor immunity [34]. Phase I and II trials utilizing nivolumab in patients with NSCLC demonstrated an increased median OS [35,36]. A subsequent phase III study by Brahmer et al. found that the OS was greater with nivolumab versus docetaxel (9.2 vs. 6.0 months). The authors reported PD-L1 expression was not a predictor of ICI efficacy, but subsequent studies demonstrated the importance of PD-L1 expression [37,38,39]. A study by Borghaei et al. compared nivolumab and docetaxel in NSCLC patients and also found improved OS in the nivolumab arm (12.2 vs. 9.4 months) [40]. The CheckMate 227 phase III trial found nivolumab plus ipilimumab led to an increased OS compared to chemotherapy in patients with NSCLC, although this trial excluded patients with untreated or symptomatic central nervous system metastases [41].

Pembrolizumab is another PD-1 inhibitor that has been used to treat advanced NSCLC. A phase II trial that evaluated pembrolizumab in patients with NSCLC or melanoma with untreated brain metastases found with at least 1% PD-L1 expression demonstrated a 29.7% brain metastases response [42]. Other studies found pembrolizumab plus pemetrexed–platinum [43] and atezolizumab plus carboplatin and etoposide [44] resulted in an improved OS and PFS in metastatic lung cancer. A recent systematic review found anti-PD-1 therapy had an intracerebral overall response rate of 16.4% with acceptable toxicity rates [45]. Hu et al. conducted a meta-analysis that assessed the impact that the status of brain metastases had on immunotherapy efficacy in lung cancer patients [46]. In this study, the authors reported that the utilization of immunotherapy resulted in a survival advantage in patients with brain metastases (OS hazard ratio, 0.72; PFS hazard ratio, 0.68). Notably, there was not a statistically significant survival advantage difference between brain metastases and non-brain-metastases patients; this finding suggested that immunotherapy benefits lung cancer patients regardless of the status of brain metastases.

### 3.2. Breast Cancer Brain Metastases

Breast cancer is the second-leading precursor to brain metastases. To date, numerous chemotherapies have demonstrated the ability to reduce the tumor size in breast cancer brain metastases [47], but SRS is typically the first-line treatment. Historically, endocrine-modulating therapies have been used to treat breast cancer (e.g., tamoxifen) but have failed to provide benefit for CNS metastases [48]. Other agents such as lapatinib [49,50] and abemaciclib [51,52] have yielded encouraging results, but further studies are needed to confirm the benefit of systemic therapy [53].

The discovery of HER-2/neu (a receptor tyrosine–protein kinase) led to the development of the monoclonal antibody trastuzumab, which has become an essential part of breast cancer management [54]. Although trastuzumab failed to have a significant effect on brain metastases, the antibody–drug conjugate trastuzumab–emtansine (T-DM1) [55] has demonstrated promising CNS penetrance in case series and small cohort studies [56,57,58,59]. The HER2CLIMB trial investigated the benefit of tucatinib, which is an oral, selective HER2 TKI [60]. In this study, patients with HER2-positive metastatic breast cancer who were previously treated with trastuzumab, pertruzumab, and trastuzumab emtasine were included. The patients were randomly assigned to receive either tucatinib or placebo in combination with trastuzumab and capectiabine. PFS at 1 year (33.1% vs. 12.3%) and OS at 2 years (44.9% vs. 26.6%) were significantly greater in the tucatinib group versus the placebo group. These findings suggested tucatinib plus trastuzumab and capectiabine may be beneficial for heavily pretreated HER2-positive metastatic breast cancer patients.

The ASCENT phase III trial compared sacituzumab govitecan with single-agent chemotherapy agents in patients with metastatic triple-negative breast cancer [61]. Sacituzumab govitecan is an antibody that targets Trop-2 conjugated to SN-38, a topoisomerase I inhibitor. The antibody–drug conjugate resulted in an improved median PFS (5.6 vs. 1.7 months) and OS (12.1 vs. 6.7 months) compared to the chemotherapy arm. The KEYNOTE-355 phase III trial compared pembrolizumab plus chemotherapy with placebo plus chemotherapy in patients with metastatic triple-negative breast cancer [62]. Patients that received pembrolizumab–chemotherapy with a combined positive score (number of PD-L1-positive cells divided by total number of tumor cells × 100) of ≥10 had a significantly improved PFS (9.7 vs. 5.6 months). Although patients with stable brain metastases were included in this study, patients with active central nervous system metastases were excluded.

Recently, the IMpassion130 phase III trial evaluated atezolizumab (PD-L1 monoclonal antibody) plus nab-paclitaxel in patients with locally advanced or metastatic triple-negative breast cancer [63]. Although there was no significant OS difference between the atezolizumab and placebo groups, there was a median survival benefit for patients with PD-L1 immune-cell-positive tumors (25.0 vs. 18.0 months). This finding suggested that routine testing for PD-L1 expression in patients with unresectable, metastatic triple-negative breast cancer may aid in identifying patients who may benefit from atezolizumab plus nab-paclitaxel. IMpassion 130 did not include patients with brain metastases, but a phase II study (NCT03483012) is examining the combination of atezolizumab and SRS for patients with triple-negative breast cancer that has spread the brain.

### 3.3. Melanoma Brain Metastases

Melanoma is the third most common cause of brain metastases [64]. Agents targeting BRAF and MEK have demonstrated efficacy, but primarily in patients with BRAF mutations (e.g., BRAFV600E, BRAFV600) [65]. The BREAK-MB trial evaluated dabrafenib in melanoma patients with brain metastases and found most asymptomatic patients with BRAF600E mutations exhibited an intracranial response [66].

Melanoma frequently metastasizes to the brain and is particularly resistant to RT and chemotherapy agents [67]. Previously, temozolomide was a common systemic therapy for melanoma patients with brain metastases, but temozolomide has limited CNS penetrance, with approximately 10% of patients experiencing an intracranial response [68]. Prospective phase II studies have evaluated the utility of ICIs in melanoma brain metastases [67,69,70]. In a phase II study, ipilimumab, a CTLA-4 antibody, demonstrated dose-dependent efficacy in advanced melanoma patients [71]. Tawbi et al. conducted a phase II trial including melanoma patients with untreated brain metastases receiving a combination of nivolumab and ipilimumab [72]. Rates of intracranial and extracranial clinical benefit (minimum six months of follow-up) were 57% and 56%, respectively. Grade 3 or 4 adverse events were reported in 55% of patients, but the safety profile was similar to melanoma patients without brain metastases. A phase II study by Long et al. enrolled patients with active melanoma brain metastases and found patients who received ipilimumab plus nivolumab had a durable response [73].

## 4. Complications Associated with Immune Checkpoint Inhibitors

In the 1990s, high-dose interleukin 2 (IL-2) was used to treat advanced melanoma and renal cell carcinoma patients [74]. High-dose IL-2 was associated with significant toxicity; downstream effects on T cells and natural killer cells led to a sepsis-like syndrome [75]. ICI agents can also lead to inflammatory adverse reactions that are triggered by autoreactive T cells, autoantibodies, and cytokines [76]. Single-agent ICI adverse events vary by the type of agent and tumor type. Previous reports noted that patients receiving ipilimumab or anti-PD-1/PD-L1 monotherapies experienced immune-related adverse events rates of 72% and 66%, respectively [77,78]. Dermatologic toxicity is among the most common adverse reactions reported in patients treated with CTLA-4 or PD-1/PD-L1 blockade [79]. Diarrhea is also a common adverse event with increased incidence in patients treated with CTLA-4 antibodies [80]. Hypophysitis has been seen in patients treated with anti-CTLA-4 antibodies and may develop from the antibodies binding to CTLA-4 in the pituitary [81]. Pneumonitis is a potentially life-threatening condition that can result from ICI therapy [82], although it is more commonly seen with PD-1 monotherapy than CTLA-4 monotherapy [83,84].

## 5. Radiation Dose and Fractionation Effects on Immune Response

There is a complex interplay between irradiation and the immune system [85]. In the late 1970s, researchers discovered that immunosuppressed mice required increased radiation doses to control fibrosarcoma [85], suggesting there is a link between RT and the immune system. Furthermore, there is increasing evidence that RT elicits a systemic immune response against tumor cells [86,87,88,89].

**Figure 1:**
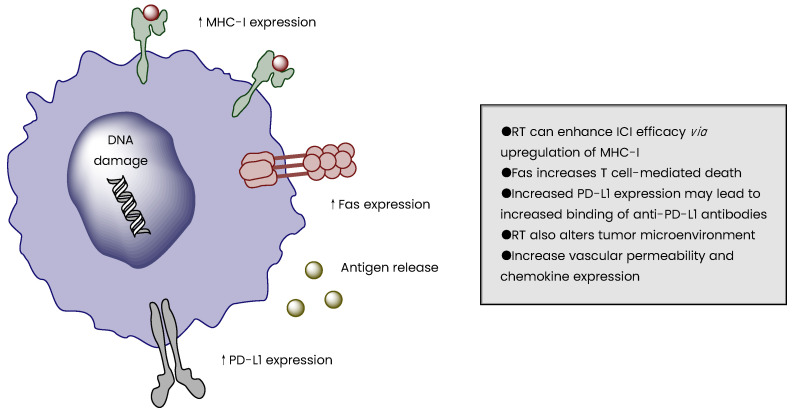
Irradiation of tumor cells leads to alterations in the immunophenotype and immunogenicity.

In the absence of RT, tumor cells evade the immune system via loss of tumor-specific antigens, but cytotoxic doses of radiation can trigger the release of these antigens, eliciting an immune response (Figure 1) [90,91,92]. The tumor-specific antigens are then presented by dendritic cells, which produces a T-cell-mediated immune response. Even at non-cytotoxic doses, RT can increase the expression of MHC-I receptors, tumor-associated molecules [93], adhesion molecules [94], and death receptors (Figure 1) [95]. This reverses the process of tumor cells downregulating the expression of cell surface molecules such as MHC-I. Another way tumors evade cell death is by downregulating Fas expression; cell surface receptors such as Fas are responsible for lymphocyte-mediated cell death [96]. Tumor irradiation can trigger the expression of Fas, thereby increasing T-cell-mediated tumor cell death (Figure 1). Radiation has also been shown to increase the expression of immune checkpoint ligands (e.g., PD-L1) and may lead to an increase in anti-PD-L1 antibodies binding tumor cells [97]. RT can also alter the tumor microenvironment and tumor-associated macrophages, induce vascular endothelial cells to express adhesion molecules (e.g., VCAM-1, E-selectin, and ICAM-1), and increase vascular permeability and chemokine expression [98,99,100,101].

In a preclinical study, Chakravarty et al. reported a synergistic interaction between RT and immunotherapy in mice inoculated with Lewis lung carcinoma [102]. In this model, mice treated with Flt3L therapy alone eventually succumbed to disease progression whereas mice that received RT (60 Gy) plus Flt3L experienced a reduction in pulmonary metastases. Furthermore, immunodeficient mice that received RT plus Flt3L did not exhibit improved survival, suggesting T cells play a key role in mediating the synergy.

Following the study by Chakravarty et al., others explored lower doses of RT in various in vitro and in vivo models [103,104]. Garnett et al. found that sublethal doses of radiation (10 or 20 Gy) greatly enhanced the susceptibility of human colon carcinoma cell lines to carcinoembryonic antigen-specific cytotoxic T lymphocyte (CTL)-mediated killing [103]. In a majority of their cell lines, there was a dose-dependent increase in Fas, MHC-I, ICAM-I, carcinoembryonic antigen, or mucin-1 expression. Chakraborty et al. demonstrated a synergistic interaction between RT and active vaccine therapy [104]. Using mice that were transgenic for the human carcinoembryonic antigen (CEA), the vaccine (vaccinia and avipox recombinants expressing CEA and T-cell costimulatory molecules) plus one dose of 6 Gy resulted in dramatic tumor volume reduction. In this study, the sublethal dose of radiation resulted in upregulation of Fas, ICAM-1, and MHC class I expression in a dose-dependent manner. The authors also recognized that radiation is typically administered in smaller daily doses, so a fractionated radiation schedule (2 Gy for 4 days) was explored. Fractionation resulted in Fas expression levels that were comparable to the single-dose regimen; 56% of the mice resolved their tumor burden.

In a B16 mouse melanoma model, Lugade et al. compared the effect of a single dose of 15 Gy to fractionated (15 Gy in 5 fractions) regimens [105]. Both regimens induced antigen presentation and priming of tumor antigen T cells, but the fractionated schedule resulted in decreased antigen presentation. Lee et al. used the same melanoma mouse model and found a single dose of 20 Gy led to decreased tumor volume and eradication of some metastases whereas a fractionated regimen (20 Gy in 4 fractions) only achieved a modest tumor reduction [106]. This report suggested that ablative RT followed by immunotherapy may lead to a synergistic effect via the generation of more cytotoxic T cells.

Schaue et al. investigated why different doses and fractionation regimens resulted in varying immune responses [107]. Using a mouse melanoma model, they reported that single-fraction doses of 7.5, 10, or 15 Gy were immunostimulatory, but a 5 Gy dose had a minimal effect. They postulated that a threshold radiation dose was necessary to switch to a pro-inflammatory response and generate IFNγ [108]. Furthermore, a single 15 Gy dose increased anti-tumor T cells and regulatory T cells, but when the mice received 15 Gy in 2 fractions, the anti-tumor T cells increased and the regulatory T cells were at their lowest level. This finding suggested that the dose and fractionation must be optimized to maximize anti-tumor T-cell activation and minimize regulatory T-cell production.

Some studies indicated that hypofractionated RT is optimal for immunomodulation [109]. Dewan et al. explored various dose-fractionation regimens to determine an optimal radiation schedule in combination with an anti-CTLA-4 antibody [109]. In breast carcinoma mouse models, various RT regimens were explored (20 Gy single dose, 24 Gy in three fractions, and 30 Gy in five fractions on consecutive days). While treatment with RT alone had no effect on secondary tumors outside the treatment field, the combination of a monoclonal antibody and fractionated RT resulted in an abscopal effect. The authors found that regression of the secondary tumor was proportional to the tumor-specific T cells. Interestingly, fractionated RT (24 Gy in 3 fractions) produced the most significant abscopal effect, but single-dose RT failed to produce this synergistic effect.

To date, there is still a debate regarding the optimal dose and fractionation needed to generate the desired immune response [110]. Pre-clinical data suggests there is not a specific dose and fractionation schedule that will elicit a pro-immunogenic effect, but that such a schedule is most likely model-dependent.

## 6. Combining Immunotherapy and Radiotherapy

RT was previously viewed as immunosuppressive when older treatment techniques utilized larger treatment fields that reduced blood cell counts [111]. Advances in the field (e.g., stereotactic body radiotherapy) have increased precision and minimized the dose to surrounding tissue, including the bone marrow. With optimal dose and fractionation, researchers believe RT may be able to counteract the immunosuppressive tumor microenvironment through a variety of mechanisms (e.g., T-cell priming, increasing MHC-I expression, enhancing tumor-associated antigen presentation, and promoting dendritic cell maturation) [112,113,114]. Furthermore, studies have suggested the sequence of RT and ICI timing is critical; a recent meta-analysis found that concurrent administration of SRS/ICIs may be associated with improved efficacy compared to sequential therapy [115]. Highlighted below are preclinical and clinical studies that explored combination strategies.

### 6.1. Preclinical Studies

One landmark study conducted by Zeng et al. found that the combination of PD-1 blockade and stereotactic radiosurgery (SRS) resulted in long-term survival in a mouse orthotopic glioblastoma model [116]. The authors reported that RT increased the expression of MHC-I, CXCL16, and ICAM in glioma cell lines, suggesting irradiation creates a pro-inflammatory response. There was also an increased CD8/regulatory T cell ratio in mice that received the combinatory treatment compared to the single modality arms.

Other murine studies that combined RT and ICIs demonstrated distant and persistent anti-tumor effects, also known as the “abscopal effect.” [117]. The abscopal effect is defined as the regression or disappearance of lesions outside of the radiation treatment field. Mole originally described the abscopal effect in 1953 [118] and Demari et al. later hypothesized the abscopal effect was likely immune-related [119]. Using a mouse mammary carcinoma model, the authors tested this theory by administering the Fms-like tyrosine kinase receptor 3 ligand (Flt3-L) to stimulate the production of dendritic cells [120]. Although Flt3-L alone was shown to cause tumor regression in other studies [121], the authors noted no effect on the primary or secondary tumors. However, Flt3-L in combination with local RT (single dose of 2 Gy) resulted in systemic anti-tumor effects even at remote disease sites. They surmised RT alone may not induce an abscopal effect due to the accumulation of immature myeloid cells that have immunosuppressive effects and a decreased number of dendritic cells [122,123].

In 2005, Demari et al. hypothesized that the combination of RT to the primary tumor and a CTLA-4 blockade may create an anti-tumor response [124]. Using poorly immunogenic mice with metastases (mammary carcinoma 4T1) as a model, the authors found that 9H10 (monoclonal antibody against CTLA-4) alone did not have an effect on the primary tumor and RT alone delayed tumor growth. Notably, the combination of RT and 9H10 resulted in improved survival rates and the inhibition of lung metastases formation.

Additional studies explored strategies that may further enhance radiation-induced immune response [125,126,127,128]. Bielecki et al. found that immunostimulatory mesoporous silica nanoparticles could deliver a stimulator of interferon genes (STING) agonist to the tumor microenvironment that reversed immunosuppression [126]. Chiang et al. developed targeted sensitization-enhanced radiotherapy (TSER) that utilized GoldenDisk as a radioenhancer and achieved selective and sustained radiosensitization effects in CD44-expressing GBM cells [127]. Chen et al. synthesized a gold-based outer-membrane vesicle (OMV) that could produce radiosensitizing and immunomodulatory effects when combined with RT [128]. The authors found that Au-OMV in combination with RT resulted in suppressed tumor growth in G261 tumor-bearing mice.

### 6.2. Clinical Studies 

#### 6.2.1. Metastatic Lung Cancer

Chen et al. conducted a retrospective analysis of NSCLC, melanoma, and renal cell carcinoma patients with brain metastases treated with SRS-SRT [129]. The patients treated with concurrent ICI compared to SRS-SRT alone had an improved median OS (24.7 vs. 12.9 months). Concurrent ICI was also associated with a decreased likelihood of developing three or more new brain metastases (odds ratio 0.377, *p* = 0.045). The overall incidence of radiation necrosis with concurrent ipilimumab was 3%, suggesting that the combination of radiosurgery and ipilimumab is relatively safe.

Schapira et al. investigated the outcomes of advanced lung cancer patients treated with SRS and PD-1 inhibitors (nivolumab, atezolizumab, and pembrolizumab) [130]. Patients receiving concurrent SRS and ICI had greater local control after 1 year, a longer OS, and reduced rates of distant brain failure after 1 year than patients treated with SRS before or after the PD-1 inhibitor. Overall, combination treatment was well tolerated; four patients required steroids (due to radiation-associated toxicity) and no patients experienced grade ≥4 toxicity.

Le et al. retrospectively evaluated local and distant control in patients with NSCLC brain metastases treated with concurrent SRS and ICI [131]. A multivariate analysis showed that concurrent therapy was not associated with improved local control but was associated with distant brain control. The authors noted that the patients receiving combination therapy were not compared to patients that received ICI alone; therefore, they were unable to determine if the decrease in distant brain failure was an additive or synergistic effect.

Waskilewski et al. compared outcomes in patients with NSCLC and brain metastases who received either adjuvant ICI or chemotherapy in combination with RT [132]. In this study, patients who received RT and chemotherapy had a significantly worse OS (11.8 vs. 23.0 months). A recent multi-center retrospective analysis evaluated outcomes of patients with advanced metastatic NSCLC treated with first-line pembrolizumab. Of the 74 patients with brain metastases who were treated with cerebral RT at diagnosis or at the initiation of pembrolizumab, 48.6% had an objective response and 24.3% had a stable disease. These findings suggested that immunotherapy agents should be considered as a treatment option for patients with NSCLC and brain metastases.

#### 6.2.2. Metastatic Melanoma

Diao et al. conducted a single-institution retrospective analysis of melanoma patients with brain metastases treated with SRS with or without ipilimumab [133]. Six months after SRS, the patients that received ipilimumab experienced a greater reduction in tumor and edema volume than the control group. The ipilimumab group also had lower rates of lesion progression but also the highest rate of lesion hemorrhage.

Another retrospective review by Rahman et al. assessed intracranial progression, OS, and radiation necrosis in melanoma patients with brain metastases who received SRS and concurrent immunotherapy (ipilimumab, pembrolizumab, and nivolumab) [134]. Although concurrent immunotherapy was associated with higher rates of intracranial progression (54.3% vs. 30.8%, *p* = 0.041), 25.7% of the patients that received the combination therapy attained ≥ 1 year intracranial progression-free survival. Toxicity analysis found the two groups had similar rates of radionecrosis, suggesting that the combination of immunotherapy and RT is relatively safe.

Kiess et al. conducted a retrospective study of melanoma brain metastases patients receiving SRS plus ipilimumab. Overall, the combination appeared to be well tolerated. They found that delivery of immunotherapy and SRS was associated with increased survival rates and locoregional control.

A retrospective study of melanoma patients with brain metastases treated with definitive radiosurgery compared ipilimumab patients with patients who did not receive ipilimumab [135]. The addition of ipilimumab was associated with an increased median survival (21.3 vs. 4.9 months) and 2-year survival (47.2% vs. 19.7%). The improved survival outcomes in the ipilimumab cohort remained significant after adjusting for performance status.

A multi-institutional retrospective analysis by An et al. assessed intracranial disease control and OS in metastatic melanoma patients treated with ipilimumab who subsequently received SRS for new brain metastases [136]. Notably, patients who delayed SRS beyond 5.5 months experienced increased rates of intracranial progression. The authors also found an absolute lymphocyte count of >1000/μL before SRS was associated with a reduced risk of intracranial recurrence (HR 0.46, *p* = 0.03).

Lastly, a retrospective study by Anderson et al. reported that patients treated with SRS and concurrent ipilimumab had a greater intracranial response than patients that received SRS without concurrent ICI (32% vs. 22%) [137]. In the same study, they reported that patients treated with SRS and concurrent pembrolizumab had significant tumor regression after treatment and >30% of the lesions had a complete response at the first follow-up scan. The authors noted that patients who exhibited a complete response had a median initial lesion size of 0.8 cm while the partial responders and stable responders had median lesion sizes of 1.0 cm and 1.1 cm, respectively. Pembrolizumab and RT appeared to have an acceptable toxicity; there were no observed grade 4 or 5 toxicities.

## 7. Ongoing Clinical Trials

Numerous preclinical and retrospective studies have demonstrated the benefit of combining RT and ICIs, but there is currently a lack of prospective clinical data. Highlighted below are ongoing phase I and II clinical trials (Table 1).

### 7.1. Metastatic Non-Small Cell Lung Cancer

A phase II clinical trial (NCT04889066) is currently evaluating the combination of durvalumab with either fractionated SRT or PULSAR (personalized ultra-fractionated stereotactic adaptive radiotherapy) in NSCLC patients with brain metastases. Eligible patients must have biopsy-proven NSCLC with ≥1% PD-L1 expression and at least one previously untreated brain metastasis (≤10 total) with no prior systemic treatment for metastatic NSCLC. This study will measure intracranial clinical benefit (primary outcome), treatment-related toxicity, and quality of life (using the Functional Assessment of Cancer Therapy–Brain questionnaire).

Another phase II study (NCT04650490) is currently evaluating the effect of timing SRS relative to ICI in patients with advanced NSCLC. The study includes one arm of immediate SRS followed by ICI (within 14 days) and another arm of immediate ICI followed by SRS. The ICI utilized is at the discretion of the treating physician. The primary outcome measure is intracranial PFS and the secondary outcomes are QOL (measured using the FACT-Br) and neurocognitive function (measured using the Hopkins Verbal Learning Test–Revised, the Trail Making Test (parts A and B), and the Controlled Oral Word Association Test).

### 7.2. Metastatic Breast Cancer

The University of North Carolina at Chapel Hill has designed a phase I/II clinical trial (NCT04711824) to evaluate the safety and efficacy of RT with olaparib followed by durvalumab for breast cancer patients with brain metastases. This study will assess treatment-related toxicity and the intracranial disease control rate as primary outcomes and intracranial response and OS as secondary outcomes. For patients to be eligible, there must be a histologically confirmed diagnosis of breast cancer (triple negative (any BRCA status) or HER2-negative status with germline or somatic BRCA mutation).

### 7.3. Metastatic Melanoma

A phase II clinical trial (NCT03340129) is studying the outcomes of melanoma patients with untreated brain metastases receiving either RT plus ipilimumab and nivolumab or ipilumumab and nivolumab alone. The study was designed to measure the neurological specific cause of death at one year (primary outcome), intra- and extracranial response, survival outcomes, toxicity, QOL, and neurocognitive function. The investigators will assess treatment-related toxicity and intra-cranial disease control rates.

### 7.4. General Brain Metastases

The Radiosurgery Dose Reduction for Brain Metastases on Immunotherapy (RADREMI) trial (NCT04047602) is a single-arm phase 1 trial that includes patients who are receiving ICI within 30 days of single-fraction SRS for 1–10 brain metastases utilizing reduced radiation dosing (18 Gy for lesions 0–2 cm, 14 Gy for lesions 2.1–3 cm, 12 Gy for lesions 3.1–4 cm) compared to the standard-of-care RTOG 90–05 established doses of 24 Gy, 18 Gy, and 15 Gy, respectively, with the goal of reducing symptomatic radiation necrosis rates following standard-of-care SRS doses (which have been reported to be as high as 20%) without compromising local control. An interim analysis showed a 6-month radiation necrosis rate of 0% and a 6-month per-lesion local control rate of 98% [144]. The University of Chicago is conducting a phase II trial (NCT04427228) for patients with brain metastases who have received ICIs (PD-1/PD-L1 and/or CTLA-4 inhibitors) within the past 6 months or will receive an ICI within the next month. This study will compare outcomes in patients receiving either multi-fraction SRS (27 Gy in 3 fractions) or single-fraction SRS (20 Gy for GTVs < 2 cm or 18 Gy for GTVs between 2 and 3 cm). The primary outcome measure is the rate of radionecrosis and the secondary outcomes are target metastases’ progression rate, OS, treatment-related toxicity, and time to intracranial failure.

## 8. Conclusions

The current landscape of brain metastases management is rapidly evolving. Historically, systemic therapy had limited utility in the treatment of brain metastases, but immunotherapy has yielded promising results. A possible key to unlock the full potential of ICIs may be leveraging the immunomodulatory effects of RT. Although there is a lack of consensus regarding the optimal RT dose and fractionation, recent preclinical studies have indicated that hypofractionated RT may be immunostimulatory. Although there have been numerous retrospective studies supporting the synergistic interaction between RT and ICIs in patients with brain metastases, the prospective data are limited. Ongoing clinical trials will provide much-needed insight that will guide clinical decisions for patients and clinicians.

## Figures and Tables

**Table 1:** Ongoing brain metastases (BMs) trials merging ICIs with RT.

Clinical Trial	Phase	Disease Site	Immune Checkpoint Inhibitor	Description	Measured Outcomes
NCT04889066 [138]	II	NSCLC BMs	Durvalumab (PD-L1 inhibitor)	Comparing durvalumab with either fractionated SRT or PULSAR	Intra-cranial benefit, toxicity, and quality of life
NCT04650490 [139]	II	NSCLC BMs	Physician’s choice of immunotherapy per standard of care	Evaluating effect of SRS timing relative to ICI	Intra-cranial PFS, quality of life, and neurocognitive outcomes
NCT04711824 [140]	I/II	Breast cancer BMs	Durvalumab (PD-L1 inhibitor)	Assessing safety and efficacy of RT with olaparib followed by durvalumab	Adverse events, intra-/extra-cranial response, and survival outcomes
NCT03340129 [141]	II	Melanoma BMs	Ipilimumab (CTLA-4 inhibitor) and nivolumab (PD-1 inhibitor)	Comparing ipilimumab and nivolumab with concurrent SRT versus ipilimumab and nivolumab alone	Specific cause of death (neurological or non-neurological), intra-/extra-cranial response, toxicity, neurocognitive/quality of life/functional status changes
NCT04047602 [142]	I	General BMs	Standard of care immunotherapy	ICI followed by single-fraction SRS at a reduced dose	Symptomatic radiation necrosis, local control, and radiographic radiation necrosis
NCT04427228 [143]	II	General BMs	Either PD-1/PD-L1 and/or CTLA-4 inhibitor within 6 months of RT	Comparing single- versus multi-fraction SRS	Acute/chronic toxicity, PFS, OS, and time to distant intra-cranial failure
